# Plasma Metabolomic Alterations Are Associated with T-Cell Senescence, Cytokine Dysregulation, and Prognosis in Elderly COVID-19 Patients

**DOI:** 10.3390/biomedicines14092118

**Published:** 2026-09-19

**Authors:** Ying Shang, Hui Zhang, Yinan Lang, Tingting Hu, Chun Chang, Liuyang Zhang

**Affiliations:** 1 Department of Respiratory and Critical Care Medicine, Peking University Third Hospital, Beijing 100191, China; 2 Research Center for Chronic Airway Diseases, Peking University Health Science Center, Beijing 100191, China; 3 Research Center of Basic Medical Sciences, Tianjin Medical University, Tianjin 300070, China; 4 Department of Human Nutrition, University of British Columbia, Vancouver, BC V6T 1Z4, Canada; 5 Department of Comprehensive Treatment, Outpatient Department, Second Medical Center of Chinese PLA General Hospital, Beijing 100853, China

**Keywords:** elderly COVID-19 patients, plasma metabolomics, T-cell senescence, cytokines, machine learning

## Abstract

**Background**: Elderly patients with COVID-19 are at increased risk of severe disease and mortality. Interactions between the plasma metabolome and the immune microenvironment may influence disease progression and outcomes; however, the associations of metabolic alterations with T-cell senescence, cytokine dysregulation, and clinical outcomes in this population remain poorly understood. **Methods:** Plasma metabolomic profiling was performed in elderly COVID-19 patients and healthy controls to identify differential metabolites and metabolic features associated with clinical outcomes. Machine learning approaches were applied to identify key metabolic features associated with 180-day mortality. Correlation analyses were performed to evaluate the associations of metabolic alterations with T-cell senescence and cytokine profiles. In vitro experiments were further performed to evaluate the effects of lysophosphatidylethanolamine (LPE) on T-cell senescence. **Results:** Plasma metabolomic profiles differed markedly between elderly COVID-19 patients and healthy controls. Distinct metabolic alterations were also observed between non-survivors and survivors, with phosphatidylethanolamine (PE) 36:3, glycochenodeoxycholic acid, and deoxycholic acid showing higher levels in non-survivors. Machine learning identified 24 key metabolites associated with 180-day mortality, primarily involving bile acid metabolism, fatty acid oxidation, and phospholipid metabolism. Dysregulated plasma phospholipid metabolism, particularly involving phosphatidylcholine (PC), lysophosphatidylcholine (LPC), phosphatidylethanolamine (PE), and lysophosphatidylethanolamine (LPE), was associated with T-cell senescence in elderly COVID-19 patients. Moreover, exogenous LPE 18:1 attenuated selected markers of T-cell senescence in vitro. Additionally, five cytokines—GRO-α, IL-6, IL-8, IP-10, and TSLP—showed higher levels in non-survivors. Metabolic alterations were associated with inflammatory cytokine profiles, particularly IP-10 and TSLP, while deoxycholic acid showed concurrent associations with IL-6, IP-10, and IL-8. **Conclusions:** Plasma metabolomic alterations were associated with T-cell senescence, inflammatory cytokine profiles, and clinical outcomes in elderly patients with COVID-19.

## 1. Introduction

The COVID-19 pandemic, caused by the SARS-CoV-2 virus, has posed a significant global health challenge, with older adults being particularly vulnerable. Epidemiological studies indicate that patients aged 65 years and older account for the majority of COVID-19-related hospitalizations and deaths, with case fatality rates significantly higher in this age group compared to younger populations [1]. Older patients are also more likely to develop severe complications, including acute respiratory distress syndrome (ARDS), multi-organ dysfunction, and mortality [2,3]. Furthermore, older adults are at increased risk of developing post-acute sequelae of SARS-CoV-2 infection (PASC), commonly referred to as “long COVID”, which is characterized by persistent symptoms such as fatigue, cognitive impairment, and dyspnea. These symptoms often last for months or even longer, significantly impacting the quality of life [4]. These clinical observations underscore the unique susceptibility of older adults to both the immediate and long-term effects of COVID-19, suggesting the involvement of distinct underlying pathophysiological mechanisms. While the pandemic’s peak has passed, COVID-19 continues to pose a significant health threat to elderly individuals, necessitating further investigation into its impact and underlying factors.

Metabolomics, a powerful tool for comprehensively analyzing small-molecule metabolites in biological samples, offers a promising approach to uncovering the biochemical alterations associated with diseases [5]. In COVID-19 research, metabolomics has been successfully utilized to identify metabolic features associated with disease severity, immune dysregulation, and organ damage. For instance, Shen et al. reported significant alterations in lipid, amino acid, and energy metabolism in COVID-19 patients, which were correlated with disease severity [6]. Metabolomic profiling has also been used to identify metabolic features associated with clinical outcomes in COVID-19, including post-acute sequelae [7]. However, studies specifically investigating the metabolic characteristics of elderly COVID-19 patients remain limited.

Biologically, aging is closely linked to immunosenescence, the progressive decline in immune function, and inflammaging, a state of chronic low-grade inflammation [8]. These age-related changes impair the ability of the immune system to mount an effective antiviral response while simultaneously exacerbating systemic inflammation, which heightens the risk of cytokine storms, organ damage, and thrombosis [9]. Our recent studies have further demonstrated that elevated levels of CD4+ T-cell senescence (Tsens) are strongly associated with immune dysfunction in severe COVID-19 cases [10]. In addition, aging is associated with significant metabolic dysregulation, including alterations in energy metabolism, lipid metabolism, and amino acid utilization. These metabolic changes may further influence the progression and recovery of disease [7].

Given the unique clinical and pathophysiological characteristics of older patients with COVID-19, this study aims to explore the plasma metabolomic profiles of this vulnerable population and characterize their associations with T-cell senescence, cytokine profiles, and clinical outcomes. These analyses may provide further insight into the interplay between systemic metabolic alterations and immune dysregulation in older patients with COVID-19.

## 2. Methods

### 2.1. Study Design and Subject Enrollment

Patients aged ≥65 years with COVID-19 were enrolled at the pulmonary intermediate care units of Peking University Third Hospital. All patients met the diagnostic criteria outlined in the 10th edition of the COVID-19 diagnosis and treatment guidelines issued by the National Health Commission of the People’s Republic of China [11]. COVID-19 diagnosis was confirmed by a positive RT-PCR test for SARS-CoV-2 from pharyngeal swabs or a positive antigen detection test from nasal swabs. Patients discharged from hospital were interviewed in our outpatient department or via telephone call at approximately a 30-day, 90-day and 180-day period after they were recruited in our study. The primary outcome of our study was 180-day mortality. Age-matched healthy controls without a history or clinical evidence of chronic respiratory diseases, metabolic disorders, or other conditions that could potentially influence metabolic profiles were also recruited.

### 2.2. Plasma Metabolomics Analysis

Plasma metabolites (100 μL plasma) were extracted by protein precipitation with cold methanol (400 μL, at a plasma-to-methanol ratio of 1:4, *v*/*v*). Metabolic profiling was performed using an untargeted LC-MS approach on a Thermo Orbitrap Exploris 480 mass spectrometer coupled with a Vanquish UHPLC system (Thermo Fisher Scientific, Waltham, MA, USA), operated in both positive and negative ion modes. MS/MS spectra were acquired using a data-dependent acquisition (ddMS2) strategy to enable structural confirmation of annotated compounds.

Raw data were processed using Compound Discoverer software (version 3.3, Thermo Fisher Scientific). Compound annotation was performed by spectral matching against the mzCloud database using the default search parameters (precursor mass tolerance: 5 ppm; fragment mass tolerance: 10 ppm; Identity Search mode), with ChemSpider used as a supplementary resource for chemical formula and structure annotation. Compounds with an mzCloud best-match score ≥ 70 were considered confidently annotated. Compounds were retained for downstream analysis only if they met the following criteria: mass error within ±5 ppm; detection in at least one pooled QC injection; peak area > 1,000,000 in at least 10 samples; peak-shape quality (Jaggedness) < 0.30 in at least 10 samples; and assignment of a valid compound annotation tag.

Solvent blanks and pooled QC samples (prepared by mixing equal volumes of plasma from all study samples) were injected at regular intervals throughout the analytical run to monitor instrument stability and background contamination. All samples were analyzed within a single analytical batch, and the injection order was randomized across experimental groups. Instrument stability was assessed based on the coefficient of variation (CV) of peak areas for compounds detected in QC samples, with a threshold of CV < 30% considered acceptable for untargeted plasma metabolomics.

Metabolites not detected in more than two-thirds of samples were excluded from further analysis; for the remaining metabolites, missing values were imputed using half of the minimum detected value across the dataset. Peak areas were normalized by total area normalization, followed by log2 transformation and Pareto scaling prior to multivariate statistical analysis. No internal standards were used, consistent with the untargeted profiling nature of this approach. The acquired data were processed and analyzed using the online platform MetaboAnalyst (version 6.0).

### 2.3. Sorting and Culture of T Cells

Peripheral blood from healthy donors, collected in EDTA-containing tubes, was diluted 1:1 with PBS. The samples were then layered onto Ficoll-Paque PREMIUM 1.073 (GE Healthcare, Chicago, IL, USA) and centrifuged at 400× *g* for 30 min at room temperature, with gentle acceleration and deceleration. After centrifugation, peripheral blood mononuclear cells (PBMCs) were isolated and thoroughly washed. T lymphocytes were further purified using magnetic bead separation with a commercial kit (STEMCELL, Vancouver, BC, Canada). The isolated T cells were cultured under continuous stimulation with immobilized anti-CD3 (1 μg/mL) and soluble anti-CD28 (2 μg/mL) in RPMI 1640 medium (Gibco, Grand Island, NY, USA), supplemented with 10% fetal calf serum (Gibco) and 1% penicillin/streptomycin (Gibco). Cellular senescence was induced by treating the cells with 50 ng/mL doxorubicin (Dox) for 48 h, with simultaneous administration of 0, 10 μg/mL, or 20 μg/mL LPE 18:1 (TargetMOI, Boston, MA, USA).

### 2.4. Flow Cytometry

For surface marker staining, leukocytes were incubated with fluorophore-conjugated antibodies at room temperature for 20 min in the dark. Senescence-associated β-galactosidase (SA-β-Gal) activity was assessed according to the manufacturer’s instructions for the commercial kit (Dojindo Molecular Technologies, Kumamoto, Japan), followed by surface marker immunostaining. Flow cytometry data were analyzed using FlowJo software (version 10). The following antibodies were used: PE-Cy7 anti-human CD4 (Biolegend, San Diego, CA, USA), APC-Cy7 anti-human CD8 (Biolegend), and PE-Dazzle594 anti-human KLRG-1 (Biolegend).

### 2.5. Statistical Analysis

Machine learning models, including least absolute shrinkage and selection operator (LASSO), random forest (RF), support vector machine (SVM), elastic-net regression (ENet), and extreme gradient boosting (XGBoost), were implemented using R software (version 4.2.2). Model performance was assessed using receiver operating characteristic (ROC) curves and the area under the curve (AUC) as measures of discrimination. General statistical analyses were conducted using GraphPad Prism 10. For normally distributed variables, comparisons between two groups were performed using Student’s *t*-test, while comparisons among multiple groups were performed using one-way ANOVA. For variables with non-normal distributions, the Mann–Whitney U test or Kruskal–Wallis test was applied. A two-sided *p*-value < 0.05 was considered statistically significant.

## 3. Results

### 3.1. Plasma Metabolomic Alterations in Elderly COVID-19 Patients

**Figure 1:**
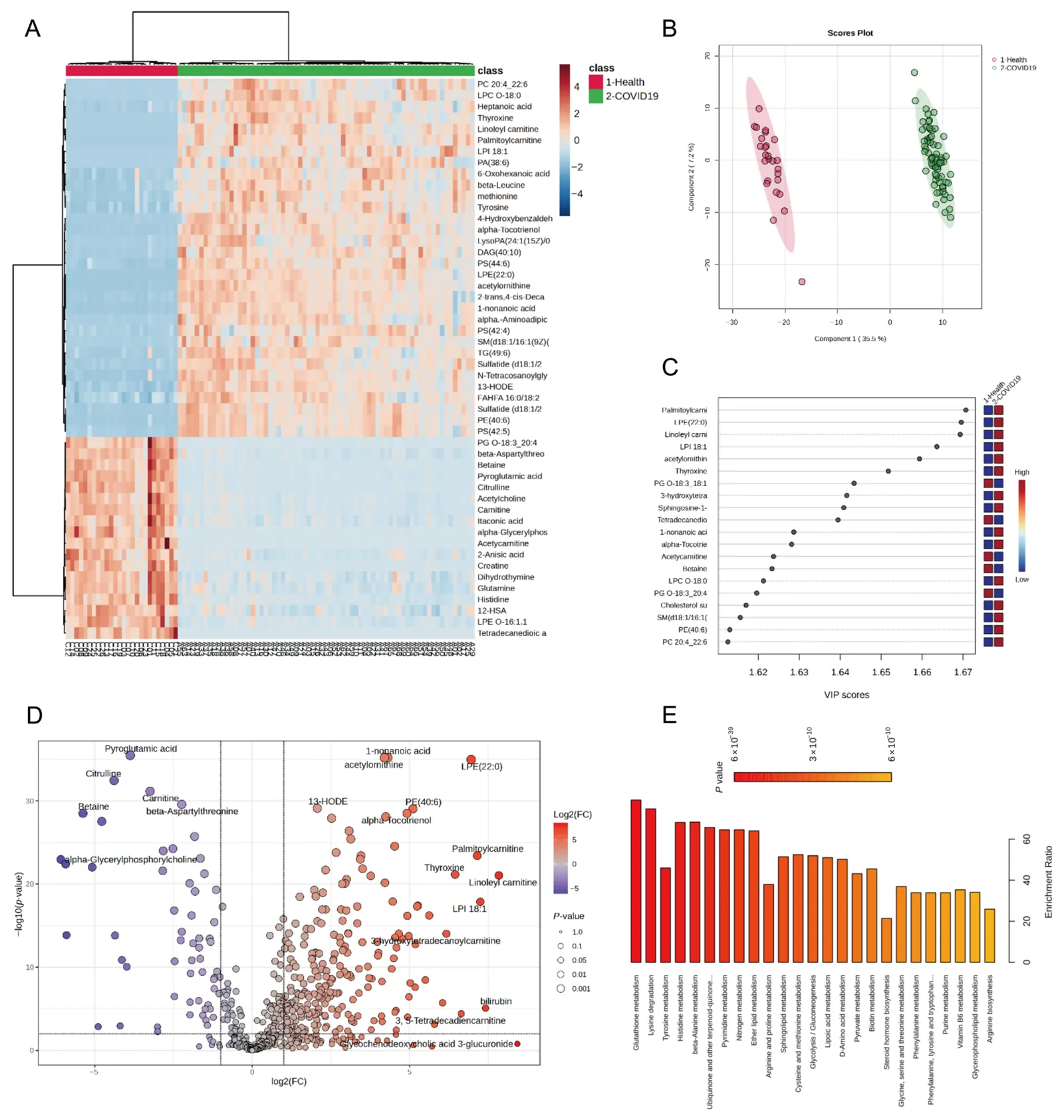
Plasma metabolic profiling analysis of elderly COVID-19 patients and healthy controls. (**A**) Heatmap of plasma metabolic profiles comparing elderly COVID-19 patients and healthy controls (top 50 metabolites with the most significant differences). The color intensity represents relative abundance: red indicates high abundance and blue indicates low abundance. Each row corresponds to a single metabolite, and each column represents an individual sample. (**B**) PLS-DA analysis of plasma metabolomic data. (**C**) VIP score plot of the PLS-DA model. (**D**) Volcano plot of differential plasma metabolites between the two groups. The horizontal axis presents log2 (fold change, COVID-19/control), and the vertical axis represents −log10 (*p* value). Red dots indicate metabolites significantly upregulated in elderly COVID-19 patients, blue dots represent significantly downregulated metabolites, and gray dots correspond to metabolites with no significant difference. The thresholds for significance were set at |fold change| > 2 and *p* < 0.05. (**E**) Functional enrichment analysis of the differential metabolites.

To investigate the characteristics of plasma metabolic profiles in elderly COVID-19 patients, we collected plasma samples from 70 elderly patients with COVID-19 and 26 age-matched healthy controls. Untargeted metabolomics analysis was performed on all samples, resulting in the identification of 596 metabolites through database matching, followed by manual correction. A heatmap depicting the relative signal intensities of the top 50 most significantly altered metabolites was generated. Clustering analysis based on these metabolites revealed significant changes in the plasma metabolic profiles of elderly COVID-19 patients (Figure 1A). Further analysis using partial least squares discriminant analysis (PLS-DA) demonstrated distinct metabolic profiles between elderly COVID-19 patients and healthy controls (Figure 1B). The variable importance in projection (VIP) score plot from the PLS-DA identified the top 20 metabolites most responsible for the overall metabolic differences (Figure 1C). To identify specific metabolites associated with the disease, univariate *t*-test analysis was conducted for each metabolite, with significant metabolites defined by a fold change > 2 and a *p*-value < 0.05 (FDR adjusted). The top 20 metabolites with the most significant changes, as determined by the *t*-test, were visualized in a volcano plot (Figure 1D). Functional enrichment analysis of these differential metabolites revealed involvement in several key metabolic pathways, including glutathione metabolism, lysine degradation, and tyrosine metabolism (Figure 1E). A Venn diagram was used to compare the top 20 metabolites identified by the VIP score plot with those identified by the *t*-test. This analysis revealed an overlap of 9 metabolites, including palmitoylcarnitine, LPE 22:0, linoleyl carnitine, LPI 18:1, thyroxine, 3-hydroxytetradecanoylcarnitine, 1-nonanoic acid, alpha-tocotrienol, and betaine. Together, these findings indicate that elderly patients with COVID-19 exhibit distinct plasma metabolic profiles compared with healthy controls.

### 3.2. Relationship Between Metabolite Features and Clinical Outcomes in Elderly COVID-19 Patients

**Figure 2:**
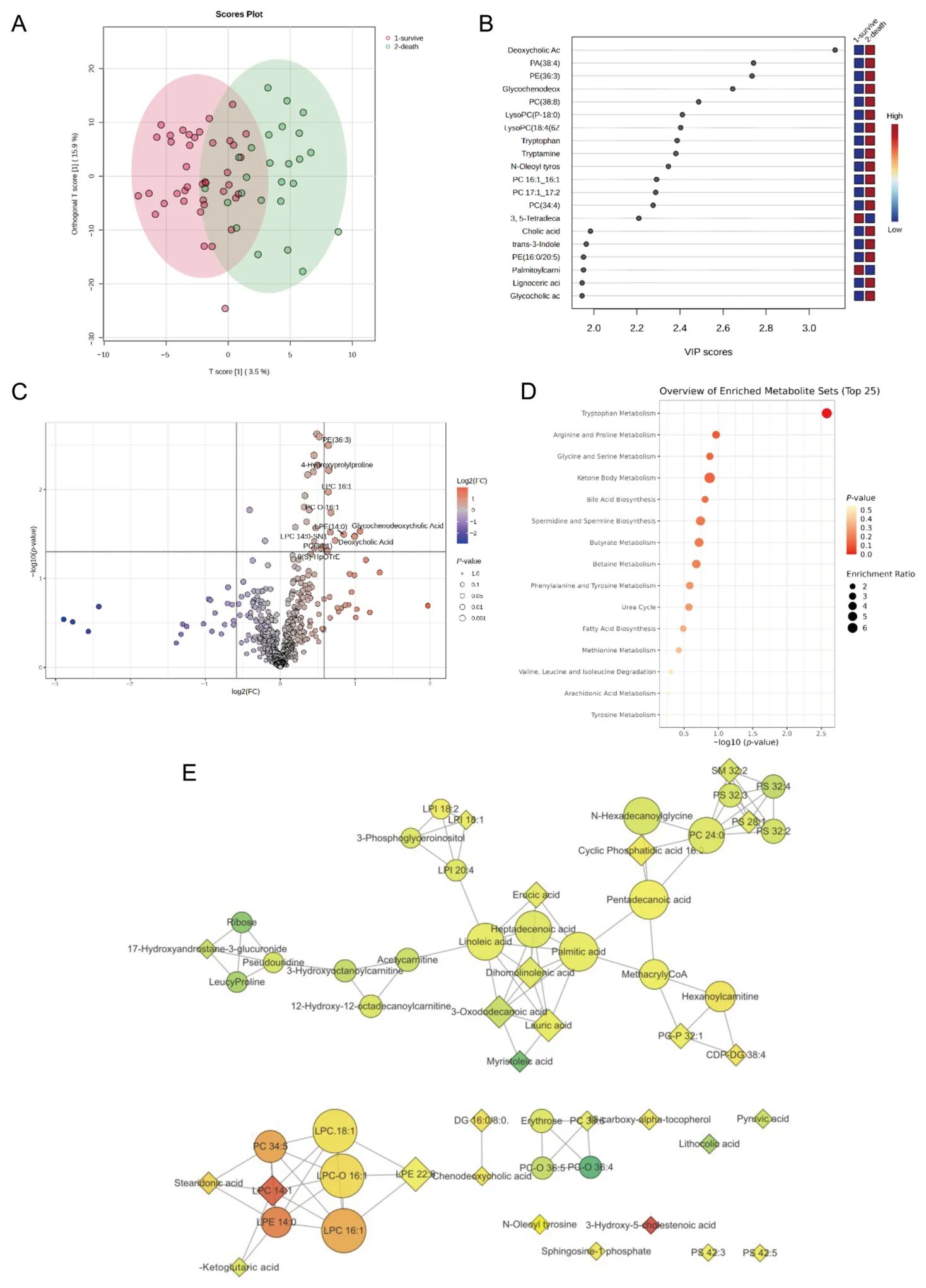
Plasma metabolomic profiling of non-survivors and survivors among elderly COVID-19 patients. (**A**) OPLS-DA analysis based on plasma metabolomic data. (**B**) VIP score plot of the OPLS-DA model. (**C**) Volcano plot of differential plasma metabolites between the two groups. The horizontal axis presents log2 (fold change, non-survivors/survivors), and the vertical axis represents −log10 (*p* value). Red dots indicate significantly upregulated metabolites in non-survivors, blue dots indicate significantly downregulated metabolites, and gray dots denote non-significant metabolites. (**D**) KEGG enrichment analysis based on changed metabolites (fold change > 1.5 and FDR-adjusted *p* < 0.1). The bubble plot displays the top 15 enriched pathways; bubble size represents the enrichment ratio, and color indicates statistical significance (*p* value). (**E**) Core metabolite interaction network. Square nodes denote seed nodes, and circular nodes denote regular nodes. Node color represents the abundance ratio (non-survivors vs. survivors), with red indicating increased levels and green indicating decreased levels; color intensity corresponds to the magnitude of change.

Clinical outcomes varied substantially among elderly COVID-19 patients. To explore whether metabolic profiles are associated with the clinical outcomes of elderly COVID-19 patients, we conducted follow-up assessments over a 180-day period and recorded mortality outcomes. Among the 70 participants in our study, 65 completed the planned follow-up, including 25 non-survivors and 40 survivors. Five participants did not complete the planned follow-up. Mortality status was used as the binary outcome variable to analyze differences in metabolomic profiles. Although the overall metabolomic differences between the “death group” and the “survival group” were not pronounced, and the discriminative ability of PLS-DA was limited, OPLS-DA analysis was still able to roughly separate the two groups (Figure 2A). The VIP score plot from the OPLS-DA model identified the top 20 metabolites contributing most to the overall differences between the groups (Figure 2B). Additionally, *t*-tests and volcano plots were used to identify metabolites that differed significantly between the two groups (Figure 2C). A Venn diagram comparing the top 20 metabolites identified by the VIP score plot with those identified by the *t*-test revealed an overlap of three metabolites: PE 36:3, glycochenodeoxycholic acid, and deoxycholic acid. To further identify metabolites significantly contributing to group classification, we applied the Significance Analysis of Microarrays (SAM) and Empirical Bayes Analysis of Microarrays (EBAM) algorithms. Next, we conducted KEGG enrichment analysis to investigate the metabolic pathways associated with these metabolites. Notably, “tryptophan metabolism” was the most highly enriched pathway, while other enriched pathways included “arginine and proline metabolism”, “glycine and serine metabolism”, “ketone body metabolism” and “bile acid biosynthesis” (Figure 2D).

To explore the relationships among metabolites in our dataset, we performed Pearson’s correlation analysis on the differential metabolites in the two groups, selecting those with correlation coefficients >0.5 or <−0.5 and FDR-adjusted *p*-values < 0.05 to construct a correlation network. The network contained 508 metabolites and 2177 interactions. To identify key nodes within this network, we used the MCODE network analysis module to extract topologically independent sub-networks. We selected the top 10 sub-networks based on MCODE scores and extracted the three metabolites with the highest connectivity from each sub-network to construct a core metabolite interaction network. Within this network, lysophospholipids (e.g., LPC 16:1, LPC 18:1, LPC-O 16:1, LPC 14:1, LPE 14:0), phospholipids (e.g., PC 34:5), fatty acids (e.g., linoleic acid, palmitic acid, erucic acid), and carnitine derivatives were significantly elevated in non-survivors. In contrast, ribose, pseudouridine, and related metabolites were significantly decreased in non-survivors (Figure 2E). These analyses identified metabolic alterations distinguishing survivors from non-survivors from multiple perspectives.

### 3.3. Machine Learning-Based Identification of Metabolic Features Associated with Prognosis in Elderly COVID-19 Patients

**Figure 3:**
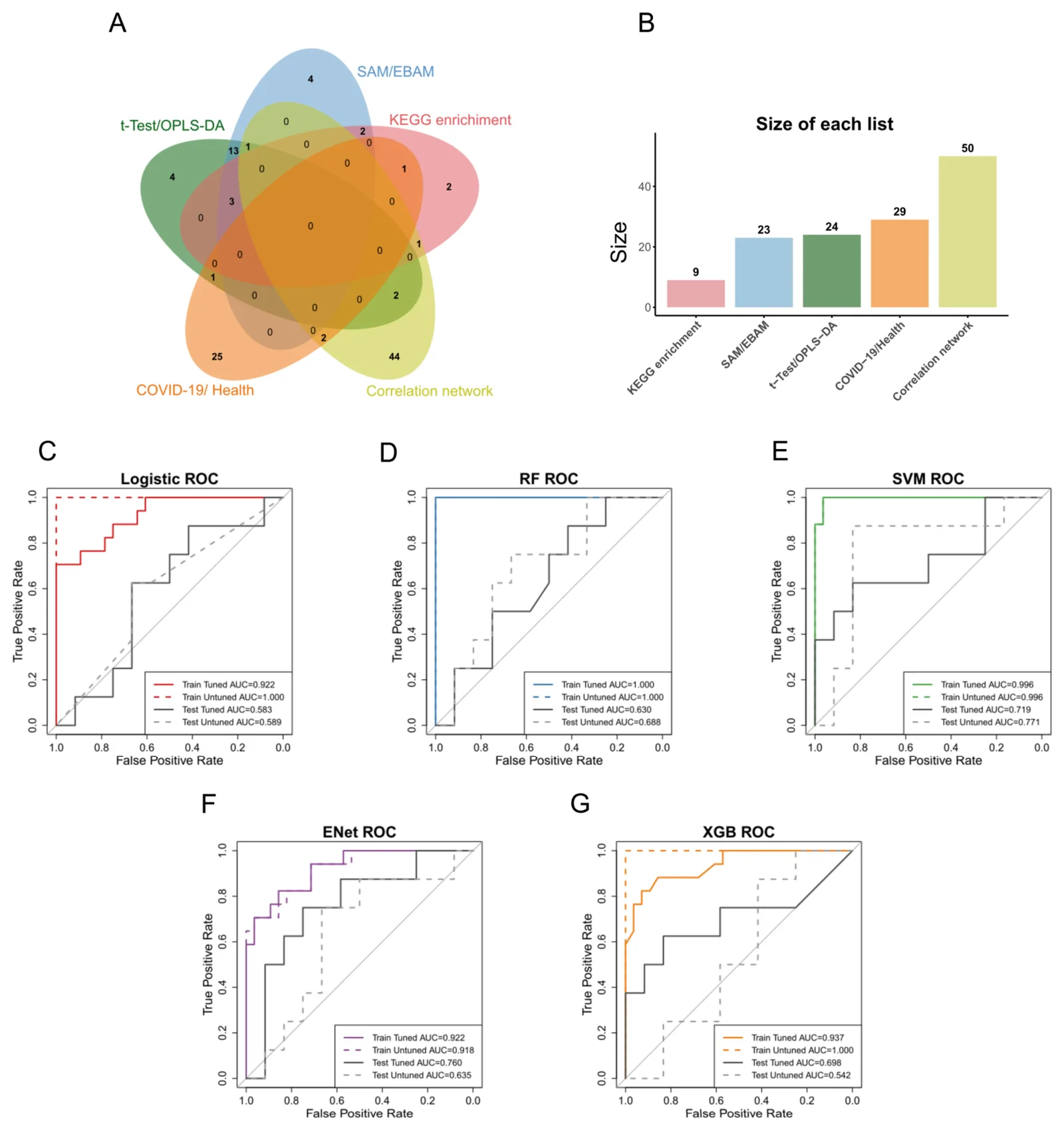
Machine learning-based identification of metabolic features associated with prognosis in elderly COVID-19 patients. (**A**) Venn diagram illustrating the overlap among candidate metabolites selected for machine learning analysis. (**B**) Number of candidate metabolites in each selection method. (**C**–**G**) ROC curves showing the discrimination of machine learning models based on the selected metabolites: (**C**) least absolute shrinkage and selection operator (LASSO) logistic regression, (**D**) random forest (RF), (**E**) support vector machine (SVM), (**F**) elastic-net (ENet) regression, and (**G**) extreme gradient boosting (XGBoost). For each model, curves are shown for the training set (colored lines: solid, tuned models; dashed, untuned models) and the test set (black lines: solid, tuned models; dashed, untuned models). AUC values are provided in the corresponding legends.

To identify metabolic features associated with 180-day mortality in elderly COVID-19 patients, multiple machine learning algorithms were applied. Prior to the machine learning screening, in order to reduce the number of features, decrease the complexity of the machine learning model, and reduce the risk of overfitting, we first performed an initial screening of the metabolites in several ways. Candidate metabolites for machine learning analysis were preselected based on the following criteria: (1) the top 20 metabolites with the lowest FDR-adjusted *p*-values from *t*-tests or the top 20 metabolites with the highest VIP scores from OPLS-DA; (2) metabolites identified by SAM or EBAM; (3) metabolites involved in the five most significantly enriched KEGG pathways; (4) metabolites within the core interaction network; and (5) differential metabolites between COVID-19 patients and healthy controls. Notably, some metabolites met multiple selection criteria. A Venn diagram was used to illustrate the overlap among these candidate metabolites (Figure 3A,B). The union of these features, comprising 105 metabolites, was used for subsequent analysis.

Five machine learning algorithms including LASSO, RF, SVM, ENet, and XGBoost were employed for the initial screening to identify metabolites associated with 180-day mortality based on the 105 candidate metabolites. Following feature selection by each algorithm, the selected features were combined to generate a union set of 24 metabolites, including carnitine, ornithine, pyruvic acid, tryptophan, tryptamine, chenodeoxycholic acid, deoxycholic acid, erucic acid, PA 38:4, PE 36:3, lithocholic acid, PC 30:1, PC 34:4, PC 38:8, PE 16:0/20:5, LPE 22:0, LysoPC (P-18:0), ribose, N-oleoyl tyrosine, 3-hydroxyoctanoylcarnitine, 4-hydroxyprolylproline, glycochenodeoxycholic acid, PC (16:1/16:1), and PC (17:1/17:2). These metabolites are mainly involved in pathways related to bile acid metabolism, carnitine and fatty acid oxidation, amino acid and tryptophan metabolism, phospholipid metabolism, and energy metabolism.

The dataset was randomly divided into training and test sets. Predictive models were constructed using the training set based on the 24 selected metabolites, and hyperparameters were optimized via cross-validation. Model performance was subsequently evaluated in the test set, and ROC curves were generated to assess and compare model discrimination across algorithms (Figure 3C–G). Most models exhibited strong performance in the training set, with AUC values exceeding 0.9, suggesting a potential risk of overfitting. Among the models, elastic-net regression achieved the best performance in the test set, with an AUC of 0.760 (Figure 3F). Candidate metabolite selection was informed by outcome-related analyses performed before the final train/test split, which may have contributed to some optimism in the estimated model performance.

### 3.4. Association Between Plasma Metabolome and T-Cell Senescence

In our previous study, increased CD4+ T-cell senescence was associated with adverse clinical outcomes in elderly COVID-19 patients; however, whether this phenotype is linked to metabolic alterations remains unclear [10]. To address this, T-cell phenotypic data were integrated with plasma metabolomic profiles for correlation analysis.

**Figure 4:**
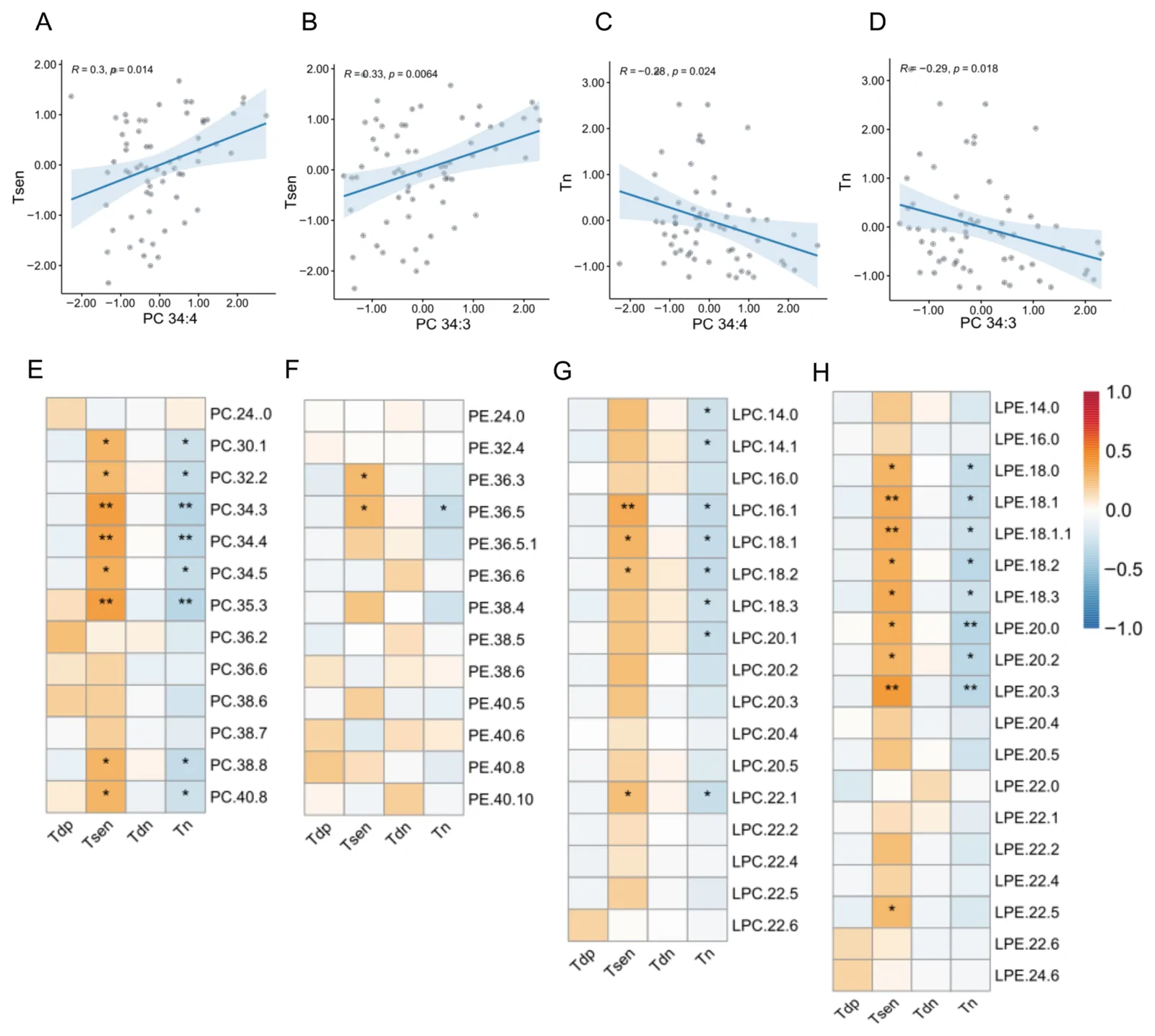
Associations between the plasma metabolome and T-cell senescence in elderly COVID-19 patients. (**A**–**D**) Correlations between representative plasma phospholipid species and proportions of Tsen or Tn. (**A**) PC 34:4 vs. Tsen proportion; (**B**) PC 34:3 vs. Tsen proportion; (**C**) PC 34:4 vs. Tn proportion; (**D**) PC 34:3 vs. Tn proportion. (**E**–**H**) Correlation heatmaps showing associations between plasma phospholipid species and T-cell subset proportions defined by CD28/CD57 expression: CD28+CD57− (Tn), CD28−CD57− (Tdn), CD28+CD57+ (Tdp), and CD28−CD57+ (Tsen). (**E**) phosphatidylcholine (PC) species; (**F**) phosphatidylethanolamine (PE) species; (**G**) lysophosphatidylcholine (LPC) species; (**H**) lysophosphatidylethanolamine (LPE) species. * *p* < 0.05, ** *p* < 0.01.

We first examined the 24 key metabolites identified from machine learning modeling and found that two phosphatidylcholine (PC) species, PC 34:3 and PC 34:4, were positively correlated with senescent T cells (Tsen) (Figure 4A–D).

Given that lipid species within the same class often exhibit coordinated changes in metabolomic and lipidomic datasets, we next extended the analysis to all annotated glycerophospholipids. Notably, in addition to PC 34:3 and PC 34:4, multiple PC and phosphatidylethanolamine (PE) species, as well as their corresponding lysophospholipids, lysophosphatidylcholine (LPC) and lysophosphatidylethanolamine (LPE), showed significant associations with T-cell senescence phenotypes. In contrast, other phospholipid classes did not exhibit significant correlations (Figure 4E–H).

**Figure 5:**
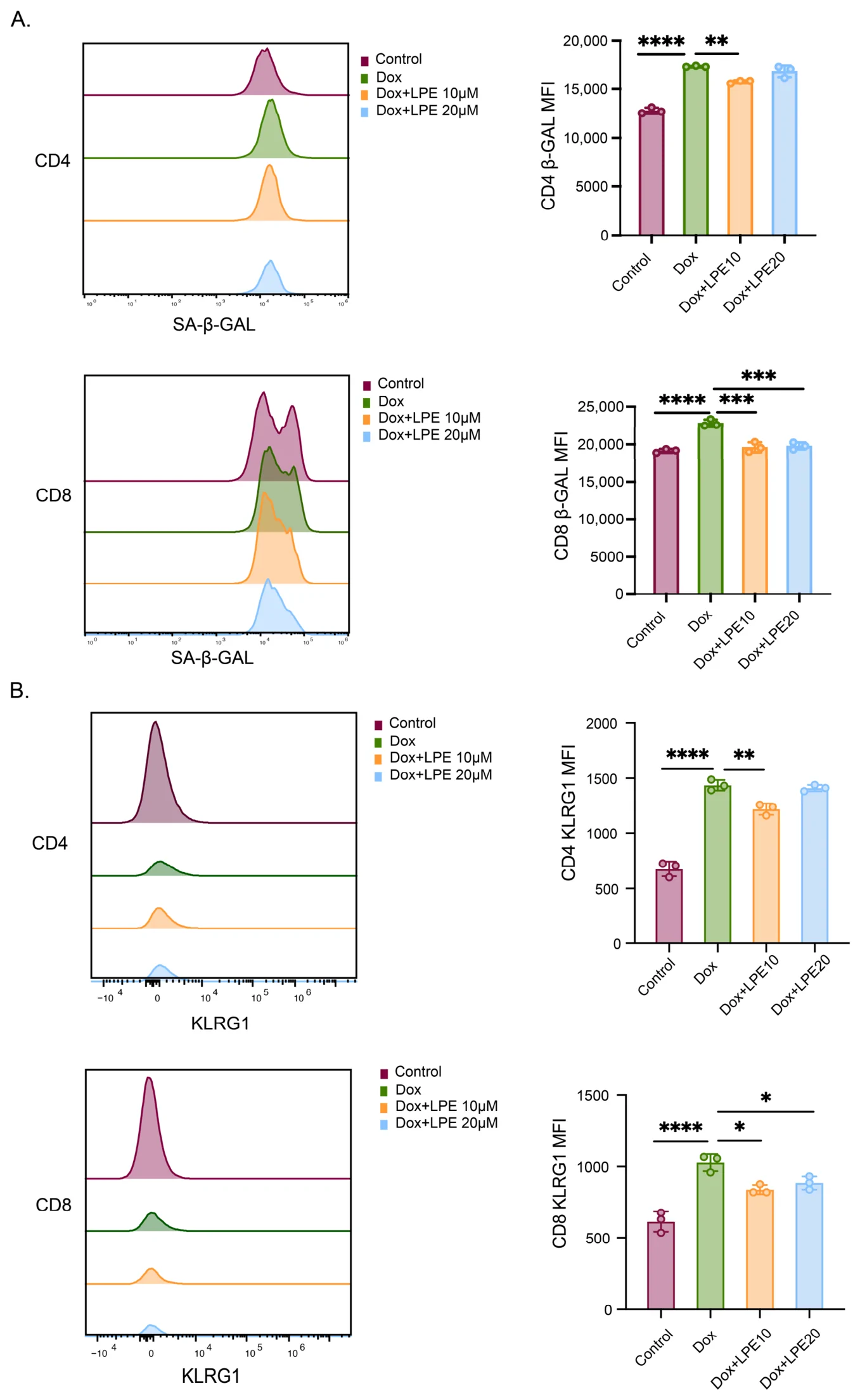
In vitro effects of LPE on T-cell senescence-associated phenotypes. (**A**) Effects of exogenous LPE 18:1 (10 μg/mL, 20 μg/mL) on SA-β-Gal expression in Dox-induced senescent CD4+ and CD8+ T cells. (**B**) Effects of exogenous LPE 18:1 (10 μg/mL, 20 μg/mL) on KLRG1 expression in Dox-induced senescent CD4+ and CD8+ T cells. * *p* < 0.05, ** *p* < 0.01, *** *p* < 0.001, **** *p* < 0.0001.

LPE 18:1 was selected as a representative phospholipid for subsequent in vitro experiments. T cells were isolated from the peripheral blood of healthy donors and induced to undergo senescence by doxorubicin treatment, followed by incubation with LPE 18:1. T-cell senescence was evaluated using canonical markers, including senescence-associated β-galactosidase (SA-β-GAL) and killer cell lectin-like receptor subfamily G member 1 (KLRG1). LPE 18:1 significantly attenuated doxorubicin-induced upregulation of SA-β-GAL (Figure 5A) and KLRG1 (Figure 5B) in both CD4+ and CD8+ T-cell subsets. These findings support an association between circulating phospholipid species and T-cell senescence and provide preliminary functional evidence for an effect of exogenous LPE 18:1 on senescence-associated phenotypes.

### 3.5. Association Between Plasma Metabolome and Cytokines

**Figure 6:**
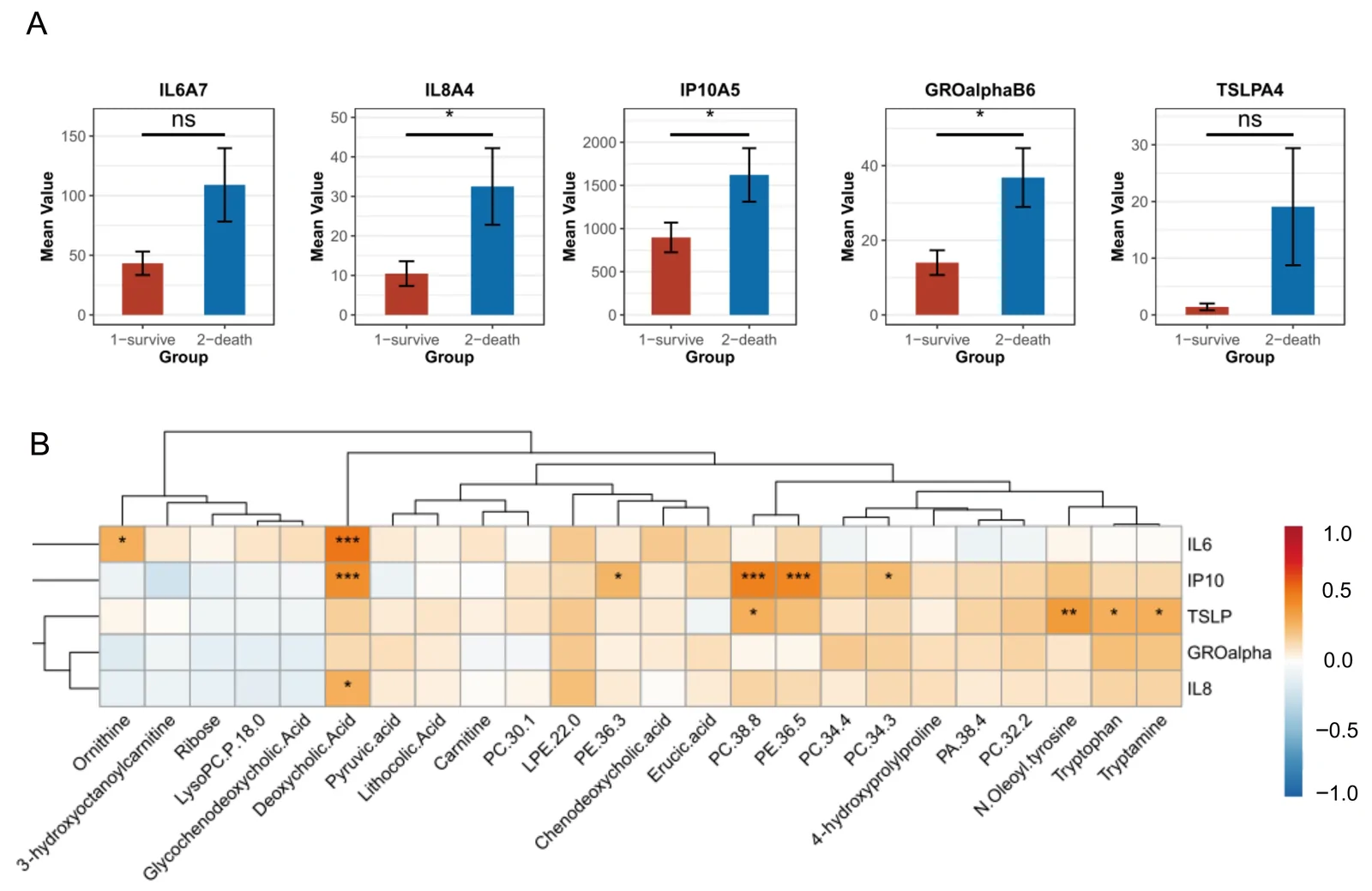
Associations between plasma cytokines and metabolites in elderly COVID-19 patients. (**A**) Comparison of plasma cytokine levels between survivors and non-survivors. (**B**) Correlation heatmap showing associations between five cytokines and the 24 key metabolites identified by machine learning models. The color scale represents the Pearson correlation coefficient (red, positive correlation; blue, negative correlation). * *p* < 0.05, ** *p* < 0.01, *** *p* < 0.001, ns, nonsignificant.

To further elucidate the relationship between metabolites and the immune microenvironment, cytokine data from our previous study were incorporated into the analysis [10]. A total of 44 cytokines were quantified using multiplex bead-based immunoassays in the same cohort. Among these, five cytokines—growth-regulated oncogene-α (GRO-α), interleukin-6 (IL-6), interleukin-8 (IL-8), interferon gamma-induced protein 10 (IP-10), and thymic stromal lymphopoietin (TSLP)—showed higher levels in non-survivors (Figure 6A).

Exploratory correlation analysis was then performed between these five cytokines and the 24 metabolites included in the machine learning models. Notably, IP-10 and TSLP showed the strongest associations with metabolic alterations (Figure 6B). Specifically, IP-10 levels were positively correlated with deoxycholic acid and several glycerophospholipid species, whereas TSLP was primarily associated with tryptophan and its related metabolites. In addition, deoxycholic acid was concurrently associated with multiple cytokines, including IL-6, IP-10, and IL-8.

## 4. Discussion

Although COVID-19 is no longer considered a global pandemic, elderly individuals remain highly susceptible to severe disease and death. Characterizing metabolic alterations in relation to immune dysfunction and disease severity may provide further insight into the biological features of severe COVID-19 in elderly patients.

In this study, untargeted metabolomics revealed profound plasma metabolic alterations in elderly COVID-19 patients, with distinct metabolic profiles between survivors and non-survivors and machine learning identifying metabolic features associated with mortality. Notably, dysregulated phospholipid species were associated with T-cell senescence and correlated with key inflammatory cytokines. These findings provide an integrated view of metabolic alterations associated with immune dysfunction and clinical outcomes in elderly patients with COVID-19.

### 4.1. Metabolomic Differences Between Elderly COVID-19 Patients and Healthy Controls

Metabolomic profiling revealed marked metabolic disturbances in elderly COVID-19 patients, particularly in amino acid metabolism and glycerophospholipid metabolism. Several metabolites, including carnitine, acylcarnitines, phosphatidylethanolamines, 1-nonanoic acid, and betaine, were significantly dysregulated, indicating broad systemic metabolic alterations in elderly patients with COVID-19. These findings are broadly consistent with previous reports describing altered plasma metabolomes in COVID-19 patients. For instance, reduced levels of betaine and citrulline have been consistently observed after infection [12,13]. Given the roles of betaine in antioxidant and anti-inflammatory processes and citrulline in nitric oxide-mediated cardiovascular protection, depletion of these metabolites may contribute to the metabolic and inflammatory disturbances observed in COVID-19 [14,15].

A notable finding in our cohort was the pattern of elevated acylcarnitines and reduced free carnitine. This pattern may be consistent with altered mitochondrial fatty acid β-oxidation. During infection, increased energetic demands promote lipid utilization; however, mitochondrial dysfunction may result in incomplete β-oxidation, leading to acylcarnitine accumulation and carnitine depletion [16,17]. In elderly individuals, this process may be further aggravated by age-associated declines in mitochondrial function and biogenesis, as well as chronic oxidative stress and low-grade inflammation [18,19]. These findings suggest that alterations in the acylcarnitine–carnitine axis may be associated with disrupted energy homeostasis in elderly COVID-19 patients.

In addition to energy metabolism, we observed significant alterations in phospholipid metabolism, with elevated levels of multiple glycerophospholipid species, including LPE, lysophosphatidylinositol (LPI), and LPC. These findings are consistent with previous studies reporting increased plasma LPC 16:1 and LPE 18:1 in COVID-19 patients [20]. Collectively, these findings indicate broad alterations in the plasma metabolome of elderly patients with COVID-19. However, the present study cannot determine whether the observed metabolic alterations are specific to COVID-19 or reflect broader metabolic consequences of acute illness, systemic inflammation, organ dysfunction, or age-related metabolic changes.

### 4.2. Metabolomic Signatures Associated with Prognosis in Elderly COVID-19 Patients

Elderly patients with COVID-19 are at high risk of adverse clinical outcomes. To investigate metabolic alterations associated with clinical outcomes, we compared plasma metabolomic profiles between survivors and non-survivors. Only a limited number of metabolites differed significantly between the two groups, with limited overlap with those altered between patients and healthy controls, suggesting that metabolic changes associated with clinical outcomes may be partially distinct from the broader metabolic perturbations induced by SARS-CoV-2 infection.

Combined OPLS-DA and univariate analyses identified phospholipids and bile acids as the major metabolic features distinguishing survivors from non-survivors. In particular, glycochenodeoxycholic acid (GCDCA) and deoxycholic acid (DCA) were significantly elevated in non-survivors, consistent with previous observations in severe COVID-19 [21]. Elevated GCDCA has been reported to induce cytotoxicity in hepatocytes and intestinal epithelial cells, promoting apoptosis and inflammatory signaling [22]. Similarly, increased DCA is associated with enhanced oxidative stress, reactive oxygen species production, and metabolic disorders such as obesity and insulin resistance [23,24]. These findings raise the possibility that altered bile acid metabolism may be associated with systemic inflammatory and metabolic disturbances in severe disease.

In addition, multiple phospholipid species, including PE, PC, LPE, and LPC, were significantly increased in non-survivors, consistent with previous studies reporting elevated circulating lysophospholipids in severe COVID-19 [20,25]. Phospholipid remodeling may contribute to disease progression by modulating immune activation, amplifying pro-inflammatory cytokine release, and disrupting membrane and endothelial homeostasis, all of which are critical features of severe COVID-19 pathology [20]. Altered phospholipid metabolism may also affect the cellular lipid environment involved in viral replication [26]. Collectively, these results suggest that coordinated dysregulation of bile acid and phospholipid metabolism is associated with adverse clinical outcomes in elderly COVID-19 patients.

To further explore the association of these metabolic alterations with prognosis, we applied a stepwise feature selection strategy integrating differential metabolites, enriched KEGG pathways, and network-derived features to reduce dimensionality and identify biologically relevant metabolic features. Following secondary feature refinement, a final 24-metabolite panel enriched in pathways related to bile acid metabolism, phospholipid metabolism, amino acid metabolism, and energy homeostasis was identified.

Models constructed using this metabolite panel demonstrated strong discrimination in the training set, although evidence of overfitting was observed in some algorithms, particularly random forest. After hyperparameter optimization, elastic-net achieved the best discrimination in the test set, with an AUC of 0.760. However, candidate metabolite selection was informed by outcome-related analyses performed before the final train/test split, which may have contributed to some optimism in the estimated model performance. In addition, the relatively small sample size and limited number of mortality events may make performance estimates based on a single random split less stable. Therefore, the observed test-set performance should be interpreted cautiously.

Our findings are consistent with previous metabolomics studies showing that fatal COVID-19 is characterized by distinct metabolic signatures, particularly involving lipid and lipoprotein metabolism [27]. Together, these findings highlight the value of integrating metabolomics with machine learning to explore metabolic features associated with adverse outcomes in elderly patients with COVID-19. Nevertheless, the 24-metabolite panel remains exploratory and requires confirmation in larger, independently validated cohorts.

### 4.3. Phospholipid Metabolism and T-Cell Senescence

In our previous study, we demonstrated that increased CD4+ T-cell senescence was associated with severe disease and poor survival in elderly COVID-19 patients and may contribute to impaired antiviral immunity and reduced vaccine responsiveness [10]. In the present study, we further investigated the metabolic features associated with this immune aging phenotype.

Correlation analysis of the 24 metabolites selected by machine learning identified several phospholipid species associated with T-cell senescence phenotypes. Extending the analysis to all detected phospholipids further revealed broad associations between phospholipid metabolism and T-cell senescence in elderly COVID-19 patients. Multiple phospholipid classes, including PC, PE, LPC, and LPE, were associated with T-cell senescence phenotypes. The consistency of these associations across different phospholipid classes suggests that altered circulating phospholipid metabolism may accompany T-cell senescence in elderly patients with COVID-19. However, given the broad involvement of phospholipid metabolism in aging, inflammation, and infection, these findings do not establish a COVID-19-specific or causal link between phospholipid alterations and T-cell senescence.

Among the phospholipid species identified in the clinical analyses, LPE 18:1 was selected as a representative phospholipid metabolite for in vitro experiments. Interestingly, despite the positive association between circulating LPE levels and T-cell senescence, exogenous LPE 18:1 attenuated senescence-associated phenotypes in the experimental T-cell model. This apparent discrepancy highlights the distinction between a circulating metabolic association and a direct cellular effect. The elevated circulating LPE observed in patients may reflect altered lipid turnover or a systemic response to infection and tissue stress rather than a direct driver of T-cell senescence. In addition, circulating LPE levels represent the net result of systemic lipid metabolism and may not reflect the local availability, cellular uptake, or downstream activity of LPE in T cells. The effects observed following exogenous LPE exposure under controlled in vitro conditions may therefore differ from those associated with endogenous LPE in the complex inflammatory and metabolic environment of elderly patients with COVID-19. Accordingly, the positive clinical association between circulating LPE and T-cell senescence should not be interpreted as evidence that LPE promotes T-cell senescence, while the in vitro findings provide only preliminary functional evidence of a potential effect of LPE 18:1 on senescence-associated T-cell phenotypes. The basis of this apparent discrepancy remains unclear and warrants further investigation in disease-relevant models.

Consistent with this interpretation, previous studies have shown that LPE supplementation can ameliorate age-related tissue dysfunction through phospholipid remodeling and improved mitochondrial homeostasis, accompanied by reduced mitochondrial oxidative stress [28]. In addition, alterations in PE- and PC-related metabolic pathways have also been reported in senescent cardiomyocytes, mesenchymal stem cells and cholangiocytes [29,30,31]. These observations provide a broader context for the potential involvement of phospholipid metabolism in cellular senescence, although they do not establish a specific role for LPE in T-cell senescence.

The molecular mechanisms linking LPE metabolism to T-cell senescence remain unclear. Emerging evidence indicates that T-cell senescence is closely intertwined with metabolic stress and mitochondrial dysfunction, which can increase ROS generation and activate stress-responsive signaling pathways, including p38 MAPK and STAT1/3, ultimately contributing to DNA damage and cell-cycle arrest [32]. Given the established role of lipid metabolism in regulating T-cell signaling, metabolic adaptation, and mitochondrial function, alterations in phospholipid composition may influence cellular processes relevant to T-cell senescence [33]. However, whether LPE 18:1 influences mitochondrial function, ROS production, or downstream p38/STAT1/3 signaling in T cells was not examined in the present study. These pathways may therefore represent potential mechanisms underlying the effects of LPE 18:1 on T-cell senescence and warrant further investigation.

### 4.4. Association Between Metabolic Phenotypes and the Cytokine Profiles in the Immune Microenvironment

Cytokines are central regulators of the immune microenvironment and are closely associated with clinical outcomes in COVID-19 [34]. Dysregulated cytokine responses, including the hyperinflammatory state commonly described as a cytokine storm, are characteristic of severe COVID-19 and have been associated with adverse clinical outcomes [34]. Beyond their pro-inflammatory roles, cytokines also critically regulate T-cell differentiation, functional maturation, and survival, thereby shaping antiviral immunity [35,36].

To further characterize immune alterations associated with poor prognosis, we analyzed multiplex cytokine profiles in elderly COVID-19 patients. Among the 44 cytokines assessed, GRO-α, IL-6, IL-8, IP-10, and TSLP showed higher levels in non-survivors, indicating an altered inflammatory cytokine profile associated with poor clinical outcomes. Consistent with previous studies, IL-6 is a key mediator of acute inflammation and cytokine storm [37], whereas IL-8 and GRO-α promote neutrophil recruitment and tissue injury and have been linked to acute respiratory distress syndrome in severe COVID-19 [38,39]. Elevated IP-10 further reflects persistent interferon-driven immune activation and inflammatory cell recruitment [40], while increased TSLP may indicate dysregulated immune homeostasis and has been associated with disease severity and prolonged hospitalization [41].

Integrative analysis of cytokines and metabolites revealed distinct patterns of associations between metabolic alterations and immune responses. In particular, IP-10 showed associations with phospholipid species, whereas TSLP was primarily associated with tryptophan and related metabolites. Notably, DCA exhibited associations with multiple cytokines, suggesting a potential role for bile acid metabolism in immune dysregulation. Given the known pro-inflammatory and oxidative properties of DCA, its elevation may be related to amplification of cytokine responses [23]; however, the direction and underlying mechanisms of these associations remain unclear.

Collectively, these findings highlight an association between metabolic alterations and immune dysregulation in elderly COVID-19 patients. The associations between phospholipids, bile acids, and cytokine profiles suggest a potential link between metabolic alterations and inflammatory responses during severe disease; however, the direction and underlying mechanisms of these relationships remain unclear.

### 4.5. Strengths and Limitations

This study has several notable strengths. First, it provides a comprehensive characterization of plasma metabolomic alterations in elderly COVID-19 patients, a clinically vulnerable yet relatively understudied population. Second, by integrating metabolomic profiles with clinical outcomes, T-cell senescence phenotypes, and cytokine signatures, this study offers a multidimensional view of metabolic and immune alterations associated with clinical outcomes. Third, the application of multiple machine learning algorithms enabled exploratory identification of a 24-metabolite panel associated with 180-day mortality, providing a framework for further evaluation of metabolic features with potential clinical relevance.

Several limitations should also be considered. First, the relatively small cohort, limited number of mortality events, and absence of an external validation cohort limit the robustness and generalizability of the metabolic features identified. Although a test set was included, the higher performance observed in the training data for some models indicates a potential risk of overfitting, and the candidate selection process before the final train/test split may have contributed to some optimism in model performance. Thus, the 24-metabolite panel should be considered exploratory rather than a validated prognostic model and requires validation in larger, independent cohorts. The absence of comprehensive clinical covariates also precluded comparison with a clinical baseline model and assessment of the incremental predictive value of the metabolic features.

Second, the clinical analyses were observational and predominantly cross-sectional, limiting causal inference. The availability of comprehensive clinical information for evaluating potential confounding factors was limited, and metabolic profiles and clinical outcomes in elderly patients with COVID-19 may be influenced by disease severity, comorbidities, organ dysfunction, nutritional status, treatment exposure, and other clinical characteristics [1,12]. Therefore, the observed associations between metabolic alterations, immune phenotypes, and 180-day mortality should be interpreted cautiously, and their independence from these potential confounding factors could not be fully evaluated. In addition, the healthy control group was selected to minimize major chronic and metabolic conditions, which may have resulted in differences in underlying health status and comorbidity burden between the two groups. Because hospitalized non-COVID-19 controls were not available, we cannot determine whether the observed metabolic alterations are specific to COVID-19 or may also reflect broader metabolic consequences of acute illness, systemic inflammation, organ dysfunction, or age-related metabolic changes [13,18,42]. Future studies including appropriately matched hospitalized patients with non-COVID-19 respiratory infections as controls will be important to further clarify the disease specificity of these metabolic changes.

Third, the in vitro experiments provide preliminary functional evidence but do not fully recapitulate the complex biological environment of elderly patients with COVID-19. The molecular mechanisms underlying the relationship between LPE metabolism and T-cell senescence were not directly investigated. Future longitudinal and mechanistic studies using disease-relevant models and comprehensive clinical characterization will be important to clarify these relationships and determine the reproducibility, disease specificity, and clinical relevance of the identified metabolic features.

## 5. Conclusions

In summary, this study identified plasma metabolic alterations associated with clinical outcomes and immune phenotypes in elderly patients with COVID-19. Multiple phospholipid species were associated with T-cell senescence, and metabolic profiles were linked to inflammatory cytokine patterns. Exogenous LPE 18:1 further attenuated senescence-associated phenotypes in T cells in vitro, providing preliminary functional evidence of its effects on T-cell senescence-associated phenotypes. These findings highlight an association between systemic metabolic alterations and immune dysfunction in elderly COVID-19 patients, while the underlying biological mechanisms remain unresolved. Further longitudinal and mechanistic studies are needed to determine the biological significance and clinical relevance of these metabolic associations.

## Data Availability

The data supporting the findings of this study are available from the corresponding author upon reasonable request.

## Abbreviations

The following abbreviations are used in this manuscript:

DCA	Deoxycholic acid
GCDCA	Glycochenodeoxycholic acid
KLRG1	Killer cell lectin-like receptor subfamily G member 1
LPE	Lysophosphatidylethanolamine
LPC	Lysophosphatidylcholine
PE	Phosphatidylethanolamine
PC	Phosphatidylcholine
SA-β-GAL	Senescence-associated β-galactosidase
